# Aerospace Environmental Health: Considerations and Countermeasures to Sustain Crew Health Through Vastly Reduced Transit Time to/From Mars

**DOI:** 10.3389/fpubh.2020.00327

**Published:** 2020-08-19

**Authors:** Annette Sobel, Robert Duncan

**Affiliations:** ^1^Texas Tech University Health Sciences Center, Lubbock, TX, United States; ^2^Texas Tech University, Lubbock, TX, United States

**Keywords:** radiation countermeasures, aerospace environmental health, bioinformatics, precision medicine, nuclear propulsion

## Abstract

The concept of implementation of environmental health protection and sustainment in aerospace environments by definition implies a One Health systems approach. One Health indicates an inherently complex, contextually interrelated system with consideration of human, animal, plant, systems engineering, and environmental health, their interrelationships, and networks. One Health implies seamless integration of subsystem co-dependencies to achieve an outcome of overall health protection for the individual. One of the most challenging aspects of space travel involves prevention, mitigation and protection from radiation injuries. While avoidance altogether is the best approach, these authors will focus on minimized exposure through limiting time in the space radiation environment in the transit to Mars and back. Implementation of the pillars of time, distance and shielding comprise ALARA, “As Low as Reasonably Achievable” (www.cdc.gov/nceh/radiation/alara.html) and is stressed in this strategy. This general overview will briefly describe the critical components of space environmental health in anticipation of increasing duration and interaction of human, animal, and plant habitation of aerospace and extreme environments into the future. Of the many considerations that could be addressed, precision medicine, and bioinformatics are the most rapidly evolving. Complex interdependencies will emerge from macro- and micro-environmental ecosystems data analysis, not yet fully comprehended or understood in the context of space health. We will conclude this contribution with suggested new countermeasure strategies gleaned through big data analytics that may protect space crew through mitigation of radiation exposure in flight.

## Introduction

This section emphasizes the importance of an integrative, holistic system of systems, i.e., a One Health,^1^ and systems engineering approach to environmental health in space. The strategy of proactively applying environmental, bioinformatics, and public health practices to human protection and interaction with aerospace environments is the main thrust of this contribution. This contribution represents an overview of outcomes-based assessments, current knowledge, and research initiatives. The final recommendations emphasize alternative strategies to mitigate environmental challenges and threats to individual and public health.

One very promising approach to environmental health protection is employment of precision medicine tools that are individualized and focused on disease prevention and health promotion *after* radiation exposure. This approach is built upon the premise that “genes + environment = health status”^2^

In the context of extreme environments such as aerospace, the most effective methodologies to promote environmental health are preventative, and many of these approaches are relatively new and largely unexplored. Programs such as the Baylor College of Medicine's Translational Institute for Space Health^3^ in partnership with NASA's Human Research Program^4^ are addressing new and emerging challenges and predictive analytics to promote and sustain space health, with a strong emphasis on environmental factors and emerging biomarkers relevant to space travelers. Some examples of recently funded and promising efforts are listed below:

- **David Howell, Ph.D**.
  Bondwell Technologies Inc., College Station, Texas
  Immobilization and stabilization of biocatalysts for efficient pharmaceutical manufacturing
- **Robert Langer, Sc.D**.
  Massachusetts Institute of Technology, Cambridge
  Just in time medications from gastrointestinal resident microbial systems
- **Karen A. McDonald, Ph.D**.
  University of California, Davis
  *A plant-based platform for “just in time” medications*.

As the practice and refinement of precision medicine, designed with the individual astronaut in mind, evolves *in situ*, specificity, predictive capacity, and knowledge will accumulate in the realm of potential applications to aerospace environmental health. Data will continue to be collected, analyzed, and assimilated regarding individual and aggregate astronaut health with associated environmental metadata. Inferences made will lead to further preventive and treatment options to ensure health sustainability for extended duration missions.

Complex systems such as extreme environments require big data analytics, simulation, test, and evaluation to ensure relevance, timeliness, and accuracy. When considering the challenge of application of precision medicine in aerospace environments, the time-critical tasks enabling health protection may become nearly insurmountable without the application of bioinformatics platforms. Specifically, the precision bioinformatics approach invokes the development of advanced tools and methodologies for understanding individualized biological data *in situ*, with corresponding pharmacotherapeutics. Although, as stated, this paper will focus on the primary concern of radiation exposure and a primary preventive approach, we also underscore the criticality of individualized protection and vulnerability assessment to mitigate unanticipated hazards.

## Environmental Concerns

### Radiation Exposure

When defining a complex system of aerospace environmental health, the primary concerns due to space radiation may be defined as follows:

1. Radiation exposure
2. Ionizing vs. non-ionizing radiation
3. Factors determining exposure
4. Countermeasures to radiation
5. Applications to life on earth.

One of the major goals of NASA's Space Radiation Project is to enable an understanding of the environmental concerns relevant to human exploration of space. By doing so, the premise to not exceed an acceptable level of risk from exposure to space radiation is implicit. This concept is similar to the guidelines established by the Nuclear Regulatory Commission, known as ALARA (*A*s *L*ow *A*s *R*easonably *A*chievable, 10 CFR, 20.1003). Space radiation is distinct from common terrestrial forms of radiation.

Humans are routinely protected from significant exposure to radiation from the sun and from space by the magnetosphere. Fluctuating levels of high-energy protons are emitted from the sun. Space radiation consists of low levels of heavy charged particles. High-energy protons and charged particles can damage both shielding materials and biological systems. The amount, or dose, of space radiation is typically low, but the effects are cumulative. Since solar activity fluctuates, the risk of radiation exposure increases with the amount of time spent in space. Extended-duration human space travel poses great concern due to this threat, among others. Resulting possible health effects include the spectrum of radiation-induced cancers, central nervous system damage, cataracts, risk of acute radiation-induced sickness, and trans-generational DNA and health effects. Methods to estimate risk are in evolution due to the limited availability of data on human exposure.

### Ionizing vs. Non-ionizing Radiation

Countermeasures to ensure protection of living and non-living systems are essential in space and traditionally employ protective measures such as PPE.

Although non-ionizing and ionizing radiation are ubiquitous and have become essential to our daily activities, each type of radiation can cause damage to living and nonliving objects, and precautions are necessary to prevent unnecessary risks.

Ionizing radiation includes alpha particles (helium atom nuclei moving at very high speeds), beta particles (high-speed electrons or positrons), gamma rays, x-rays, and galactic cosmic radiation (GCR). Examples of non-ionizing radiation include radio frequencies, microwaves, infrared, visible light, and ultraviolet light.

On a relative scale, alpha particles (the nuclei of the helium atom) cause more damage to humans and other biological systems than beta particles, gamma rays, and x-rays. The bioeffects for a given absorbed dose of alpha particle deposition energy is thousands of times more effective and damaging.

The GCR is a dominant source of radiation that must be anticipated and planned for in all extended duration space missions. GCR is experienced on all space missions and causes more bioeffects than solar particles due to the difficulty to shield against.

GCR consists of heavy, high-energy ions of elements that have had all their electrons stripped away as they have transited through the galaxy at nearly the speed of light. These particles can cause the ionization of atoms as they pass through matter and can pass relatively unimpeded through a typical spacecraft or the skin of a space traveler. The intensity of these particles is affected by the Sun's magnetic field. The average highest intensity correlates with minimum sunspots, indicating the nadir of the Sun's magnetic field and deflection minima^5^.

### Factors Determining the Amount of Radiation Exposure

Radiation exposure is quantified using a number of composite metrics. When evaluating metrics of space health effects, the following factors are considered: the energy of the radiation absorbed, the amount of radiation in the environment, and the energy of the radiation itself. These properties comprise the total radiation “dose equivalent.”

There are three main factors that determine the amount of radiation exposure and bioeffects. These factors include the following:

- Altitude above the Earth—at higher altitudes, the Earth's magnetic field is weaker, so there is less protection against ionizing particles, and spacecraft pass through the trapped radiation belts more often. During extended duration space travel, this will be a relatively small consideration. However, altitude combined with orbital inclination and proximity to the Earth's poles (maxima of ionizing particles dues to ionizing particles) correlate with increased radiation.
- Solar cycle—the Sun's 11-year cycle, culminating in a peak in the number and intensity of solar flares, especially during periods when there are numerous sunspots.
- Individual susceptibility—this is an active area of research, and not well-understood. This field holds much promise for determining effective countermeasures from radiation bioeffects and applicability of precision medicine solutions to disease states and health maintenance^5^.

### Space Weather

Space weather is defined as the ionizing radiation environment that is encountered in space due primarily to the flux of charged particles and high-energy photons from the Sun and from other galactic and deep space sources. The Earth is protected from this radiation flux by its very thin atmosphere and by its much wider magnetosphere that extends roughly 50,000 miles toward the Sun and well-beyond that on the dark side of the Earth and, hence, downstream from the solar wind^6^ This level of ionizing radiation is measured and characterized under the auspices of the National Oceanographic and Atmospheric Administration (NOAA) within their Space Weather Prediction Center (SWPC). Ionizing radiation data are obtained by numerous satellites, including the GPS satellite constellation that orbits at 12,550 miles above the Earth (https://www.gps.gov/systems/gps/space/), the GOES satellites in the geosynchronous orbit at 22,300 miles above the Earth (http://ww2010.atmos.uiuc.edu/(Gh)/guides/rs/sat/goes/home.rxml), and the NOAA DISCOVR observatory that is located at the Lagrangean “L1” point that is ~1 million miles from the Earth in the direction toward the Sun (https://www.ngdc.noaa.gov/dscovr/portal/index.html#/). Data from these sources and optical sunspot data from the terrestrial Sunspot Solar Observatory (https://en.wikipedia.org/wiki/Sunspot_Solar_Observatory) are combined to prepare weekly space weather forecasts ranging out to 27 days by NOAA and out to 45 days by the US Air Force. The NOAA Space Weather Prediction Center also provides ongoing characterization of the space weather radiation environment in an eight-point scale, ranging from “Quiet” to “Extreme Storm.” Many other facilities exist throughout the world for characterization, analysis, and predictions of the space radiation environment, such as the Geophysical Institute at the University of Alaska in Fairbanks^7^ The actual radiation dose that a crew would receive from a space weather storm depends strongly on the amount of shielding between the crew and the radiation flux from the storm. Counterintuitively, inadequate shielding often results in larger radiation exposure to the crew than no shielding at all, due to the cascading of highly energetic charged particles into showers of much lower energy-charged particle radiation jets. But the level of incoming ionizing radiation on the crew's spacecraft may be inferred from the level of the space weather radiation that is detected, as described above.

### Anticipating Extreme Environments: Human Mission to Mars

The Martian atmosphere is uninhabitable for humans and consists of the following constituents: carbon dioxide: 95.32%; nitrogen: 2.7%; argon: 1.6%; oxygen: 0.13%; and carbon monoxide: 0.08%. Also, minor amounts of water, nitrogen oxide, neon, hydrogen–deuterium–oxygen, krypton, and xenon are present^8^.

In addition, Mars is far colder than Earth due to its thinner atmosphere and further distance from the Sun. It has an average temperature of about −80°F (−60°C), with a variability from −195°F (−125°C) near the poles during the winter to as much as a comfortable 70°F (20°C) at midday near the equator. The thin atmosphere of Mars does accommodate weather, however.

Supposedly, the coupling of Mars' light gravitational field to its lack of global magnetic field left the atmosphere susceptible to pressure from solar wind and the constant stream of particles coming from the Sun. For over millions of years, solar winds caused atmospheric stripping. The etiology of the Mars atmosphere is being assessed by NASA's MAVEN (Mars Atmosphere and Volatile Evolution (MAVEN) mission^4^. Unfortunately, for humans, there is also widespread radiation at its surface. The radiation exposure would not prevent Mars exploration. However, analysis of bioeffects by the Curiosity rover concluded that a single mission to Mars is comparable for radiation tolerance guidelines for astronauts for the European Space Agency, although it does exceed the published NASA tolerance guidelines.

In addition to being plagued by Martian radiation hazards, environmental hazards on the planet include giant oxidized iron dust storms, which precipitate routinely. Peak amounts of dust occur in the northern latitudes during fall and winter, and nadir in spring seasons.

### Space Radiation and Countermeasures to Radiation Hazards

Limiting the time outside of personal protective gear and activities external to protective enclosures is one approach to mitigation of all-source hazards exposure, including radiation exposure. However, as discussed, the preventative approach to exposure is optimal to ensure protective measures outside of the Earth's atmosphere. Solar activity will primarily determine the total amount of radiation that astronauts receive, their location with respect to planetary magnetic fields, and protective measures in place. Radiation exposure for International Space Station astronauts is estimated at an annualized rate of 20–40 rems (200–400 mSv). The average dose-equivalent rate observed on a previous Space Shuttle mission was 3.9 μSv/h, with the highest rate at 96 μSv/h, which appeared to have occurred while the Shuttle was in the South Atlantic Anomaly region of Earth's magnetic field (1 Sv = 1,000 mSv = 1,000,000 μSv)^9^ The estimated Mars mission is 1,200 mSv over a 3-year period.

Historically, the crews are exposed to an average range of 80–160 mSv for a 6-month stay on the ISS at solar maximum (the time period with the maximum number of sunspots and a maximum solar magnetic field to deflect the particles) and solar minimum (the period with the minimum number of sunspots and a minimum solar magnetic field), respectively. Although the type of radiation is different, 1 mSv of space radiation is approximately equivalent to receiving three chest x-rays. Twice this annual amount of background radiation is received on Earth^5^.

An additional environmental concern during long duration space flight is severe bone loss. Microgravity environments result in an average loss of 1–2% of bone mineral density per month. For missions to Mars and beyond, bone loss requires specific countermeasures. The effects will be primarily experienced upon return to Earth when fragile bones will readily fracture. At this time, it is unknown whether this phenomenon is self-limiting^10^ The most effective countermeasure to bone loss attributable to microgravity conditions is weight-bearing exercise (https://science.nasa.gov/science-news/science-at-nasa/2001/ast01oct_1/).

## Discussion: Sustaining Aerospace Environmental Health With Space Radiation Exposure

The most significant contributor to sustainment of space environmental health protection is mitigation of radiation exposure during transit from Earth to Mars. Using a traditional approach for transit from Earth to Mars, a mission consists of the estimated shortest cruise time of a 180-day cruise to Mars (typically 35 million miles away from Earth at the closest point), a 500-day-stay planetary mission, and a 180-day return flight to Earth, which would result in a cumulative radiation dose range between 0.66 and 1.01 Sv, based on measurements by Curiosity's Radiation Assessment Detector (RAD) instrument (www.space.com).

As discussed above, the current plans for delivering astronauts to Mars from Earth using conventional chemical propulsion will require 180 days, and a prompt return flight would be of roughly comparable duration. The adverse health issues resulting from long exposures to radiation, as discussed above, could be avoided if a much more energetic propulsion system were utilized. Here we discuss the use of a nuclear-powered rocket for the mission to Mars. Such a propulsion system would permit a period of only between 2 and 4 days to complete transit from the Earth to Mars, as described below. A short duration mission would greatly reduce the time of exposure of the crew to the harsh radiation environment that exists outside of the Earth's atmosphere and magnetosphere, and it would permit the use of space weather forecasts to avoid periods of high-charged particle radiation flux during the mission. Currently, the NOAA Space Weather Prediction Center provides up to a 27-day forecast, and the US Air Force provides up to a 45-day space weather forecast. Hence, the space radiation environment is predictable during the short-duration missions that employ nuclear propulsion but entirely unpredictable for the much longer periods that could readily exceed 1 year if conventional chemical propellants were employed instead.

In this example, we assume that the rocket accelerates with a constant acceleration *a* for half of the 50 million-mile trip, and then de-accelerates with the same magnitude of acceleration for the second half of the journey, leaving the spacecraft and crew at the orbital velocity of Mars. If *a* = *g* = 9.80 m/s^2^, then the trip would require only 2.10 days to complete, and the crew would remain under the same Earth-based acceleration throughout the short journey. This, and the much shorter exposure to interplanetary radiation, would place the crew at much less health risk and would require less robust traditional countermeasures.

Assuming that the conversion of the reactor output energy to the kinetic energy of the spacecraft at its midpoint was 50% efficient during this acceleration phase, as discussed below, then such a fast transit would require a continuous power output of 17 GW throughout the trip, assuming a spacecraft mass of 1,000 kg. A maximum speed relative to the Earth of 2.0 million miles per hour, which is ~0.3% of the speed of light, would be achieved at the half-way point. If the acceleration used differed from *g*, then the time required to transit the distance to Mars would scale as a^−1/2^, and the steady reactor power output required would scale as a^3/2^. Hence, if the same distance to Mars (50 million miles) was spanned using a steady acceleration magnitude of *g*/2, then the power required would decrease to 35% of 17 GW = 5.95 GW, while the time required to make the trip would increase to 3 days. At a constant acceleration of 0.25*g*, then the power required would further reduce to 2.08 GW, and the duration of the trip would increase to just over 4 days. Finally, at a constant acceleration of 0.125*g*, the power output required would be 729 MW, and the duration would be 6 days.

**Figure 1:**
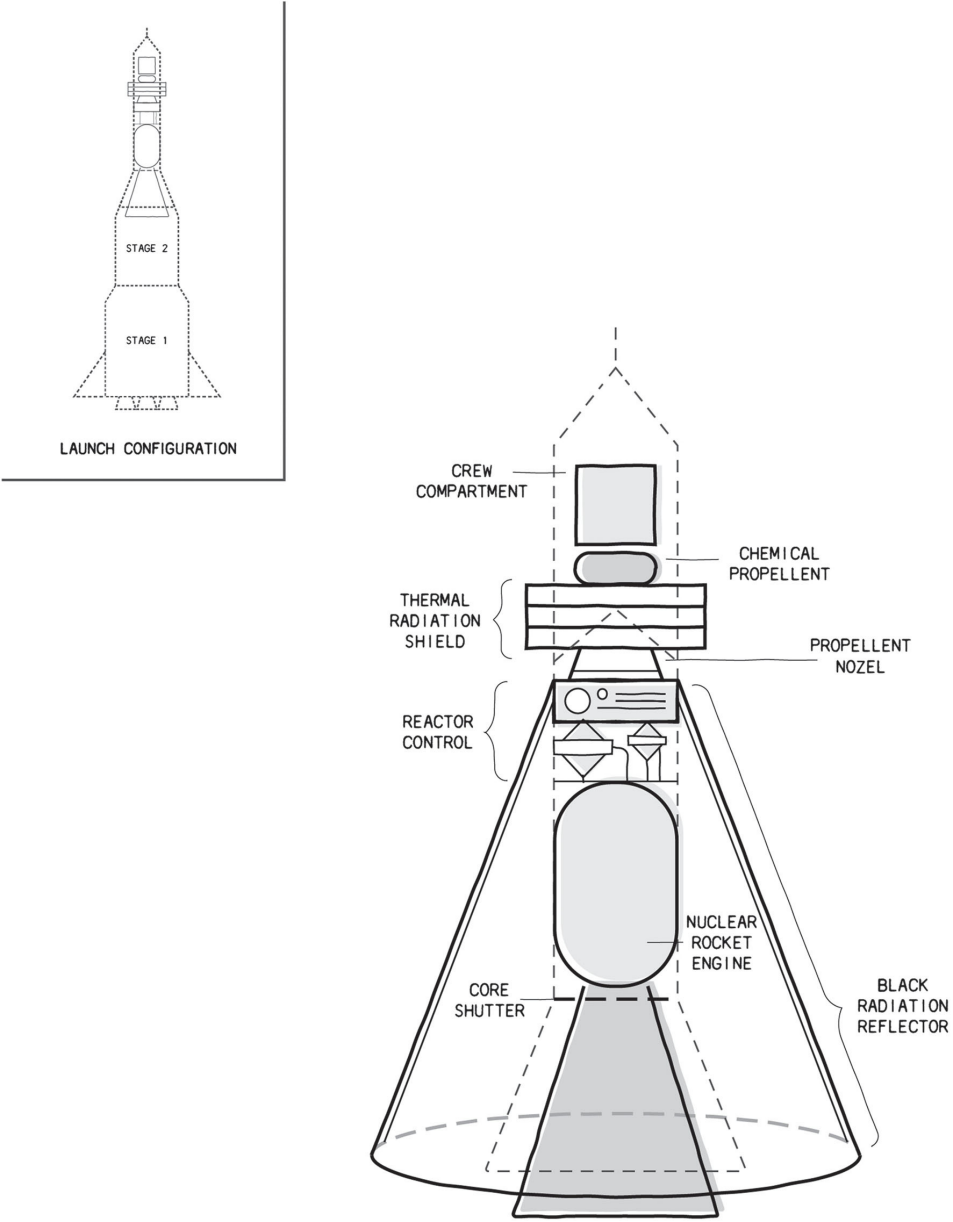
Concept of the nuclear rocket, which will greatly reduce the crew's exposure to ionizing radiation by reducing the transit time to/from Mars to less than a week. The payload/crew area is located in an excursion module that uses conventional propellant, and that will detach from the nuclear rocket for descent to Mars' surface, and to re-enter Earth's orbit and atmosphere. The nuclear section will include a very hot nuclear reactor, with a columnated ejection port, and an advanced thermal and particle radiation shield to protect the crew. The nuclear portion will be activated only when the rocket has been conventionally propelled to well-above Earth's atmosphere, at about 100,000 miles from Earth. A radiation reflector will be deployed from alongside the spacecraft using a twisting radial actuator, once the spacecraft is well-away from Earth, and before the nuclear section is taken critical. As discussed, other forms of radiation protection may be added; however, the nuclear rocket approach will greatly reduce primary exposure by greatly reducing transit times to/from Mars.

The conceptual design of the spacecraft is displayed in Figure 1. Unlike terrestrial nuclear reactors, this reactor will discharge its fission reaction products out the back of the open-core reactor. A conventional chemical propellant will be used to launch this spacecraft to about 100,000 miles from Earth, at which point the reactor core will be activated to produce its designed output power, and the exhaust shutter will be opened to expose the core reaction to the opposite direction of the spacecraft's travel. Notice above that the conversion efficiency of the total reactor output energy to the kinetic energy of the spacecraft at the half-way point of the journey was assumed to be 50%, which is supported by basic design estimates. This number will increase as the momentum of the nuclear reaction products becomes columnated more effectively to the opposite direction of the spacecraft's motion, resulting in more momentum transfer to the spacecraft. Reflectors, and other technology such as thermoelectric conversion materials, may be used to convert the resulting thermal gradient to an electrical potential that may be used to accelerate the positive ions from the nuclear reactions toward the rear of the spacecraft, and the large external reflector shown in Figure 1, which would deploy before initiating nuclear propulsion, would be used to reflect the radiation from the hot reactor assembly in the direction opposite to the spacecraft's motion. Once deployed, using a radial twisting actuator, this radiation reflector would never be retracted. The nuclear propulsion section would remain on the Mars orbit while the crew section detaches and makes the excursion to the surface of Mars. The crew section would later dock again with the nuclear portion for the return trip to Earth. The crew section would make its final detachment from the nuclear section during its de-acceleration phase, at a distance of about 100,000 miles from Earth. The crew section would then re-enter the Earth orbit, and then the atmosphere, while the nuclear rocket would be left on a disposal trajectory directly toward the Sun. The design of such a nuclear rocket will be a technical challenge, since all components, most notably the fuel composition and the fuel cycle design, will need to be developed.

## Author Contributions

All authors contributed to the article and approved the submitted version.

## Conflict of Interest

The authors declare that the research was conducted in the absence of any commercial or financial relationships that could be construed as a potential conflict of interest.

## Footnotes

^1^http://www.onehealthinitiative.com

^2^https://learn.genetics.utah.edu/content/precision/intro/

^3^www.bcm.edu

^4^www.nasa.gov

^5^www.spaceflight.nasa.gov/spacenews/factsheets/pdfs/radiation.pdf

^6^http://cse.ssl.berkeley.edu/stereo_solarwind/science_space_weather.html

^7^https://www.gi.alaska.edu/monitors/aurora-forecast

^8^www.space.com

^9^http://www.nasa.gov/mission_pages/station/science/experiments/BBND.html

^10^https://www.asc-csa.gc.ca/eng/sciences/osm/bones.asp

